# Investigating endocrine‐disrupting properties of chemicals in fish and amphibians: Opportunities to apply the 3Rs

**DOI:** 10.1002/ieam.4497

**Published:** 2021-08-18

**Authors:** Natalie Burden, Michelle R. Embry, Thomas H. Hutchinson, Scott G. Lynn, Samuel K. Maynard, Constance A. Mitchell, Francesca Pellizzato, Fiona Sewell, Karen L. Thorpe, Lennart Weltje, James R. Wheeler

**Affiliations:** ^1^ NC3Rs London UK; ^2^ Health and Environmental Sciences Institute (HESI) Washington DC USA; ^3^ School of Geography, Earth & Environmental Sciences University of Plymouth Plymouth UK; ^4^ US Environmental Protection Agency (EPA) Office of Science Coordination and Policy Washington DC USA; ^5^ AstraZeneca, Global Sustainability Cambridge UK; ^6^ Hazard Assessment European Chemicals Agency (ECHA) Helsinki Finland; ^7^ Centre for Chemical Safety and Stewardship Fera Science Ltd. York UK; ^8^ BASF SE, Agricultural Solutions−Ecotoxicology Limburgerhof Germany; ^9^ Shell Health, Shell International B.V. The Hague The Netherlands; ^10^ Present address: US Environmental Protection Agency (EPA) Office of Pesticide Programs Washington DC USA

**Keywords:** 3Rs, Animal alternatives, Aquatic toxicity, New approach methodologies (NAMs), Safety assessment

## Abstract

Many regulations are beginning to explicitly require investigation of a chemical's endocrine‐disrupting properties as a part of the safety assessment process for substances already on or about to be placed on the market. Different jurisdictions are applying distinct approaches. However, all share a common theme requiring testing for endocrine activity and adverse effects, typically involving in vitro and in vivo assays on selected endocrine pathways. For ecotoxicological evaluation, in vivo assays can be performed across various animal species, including mammals, amphibians, and fish. Results indicating activity (i.e., that a test substance may interact with the endocrine system) from in vivo screens usually trigger further higher‐tier in vivo assays. Higher‐tier assays provide data on adverse effects on relevant endpoints over more extensive parts of the organism's life cycle. Both in vivo screening and higher‐tier assays are animal‐ and resource‐intensive and can be technically challenging to conduct. Testing large numbers of chemicals will inevitably result in the use of large numbers of animals, contradicting stipulations set out within many regulatory frameworks that animal studies be conducted as a last resort. Improved strategies are urgently required. In February 2020, the UK's National Centre for the 3Rs and the Health and Environmental Sciences Institute hosted a workshop (“Investigating Endocrine Disrupting Properties in Fish and Amphibians: Opportunities to Apply the 3Rs”). Over 50 delegates attended from North America and Europe, across academia, laboratories, and consultancies, regulatory agencies, and industry. Challenges and opportunities in applying refinement and reduction approaches within the current animal test guidelines were discussed, and utilization of replacement and/or new approach methodologies, including in silico, in vitro, and embryo models, was explored. Efforts and activities needed to enable application of 3Rs approaches in practice were also identified. This article provides an overview of the workshop discussions and sets priority areas for follow‐up. *Integr Environ Assess Manag* 2022;18:442–458. © 2021 The Authors. *Integrated Environmental Assessment and Management* published by Wiley Periodicals LLC on behalf of Society of Environmental Toxicology & Chemistry (SETAC).

## KEY POINTS

- In vivo screening and higher‐tier assays to evaluate a chemical's potential endocrine‐disrupting properties and potential impacts on ecosystems are animal‐ and resource‐intensive and can be technically challenging to conduct.
- There has been much recent work to develop new approach methodologies (NAMs)—in silico and in vitro approaches—that can be applied in this space.
- An understanding of the opportunities and challenges with the current in vivo approaches, coupled with the ongoing efforts to expand the development, use, and application of NAMs, will help focus short‐ and long‐term goals.
- International, multisector science‐based activities provide the framework to improve ecological safety as it relates to compounds with endocrine‐disrupting properties.

## BACKGROUND

An endocrine‐disrupting chemical (EDC) is defined as “an exogenous substance or mixture that alters function(s) of the endocrine system and consequently causes adverse health effects in an intact organism, or its progeny, or (sub)populations” (WHO/IPCS, 2002). There are concerns regarding the potential for chemicals to cause endocrine disruption (ED) in humans and wildlife populations. Multiple regional chemical regulations now explicitly require that ED properties be investigated as part of the approval process. Testing requirements currently range from mandated testing for all substances, exposure‐based prioritization programs, and concern‐triggered testing. The approaches implemented vary by jurisdiction; however, all share common themes of testing for endocrine activity and/or adverse effects (cf. ECHA/EFSA, 2018; Japan Ministry of the Environment, 2010; USEPA, 2020).

Testing for endocrine activity (i.e., alteration of the endocrine system potentially leading to adverse effects) usually involves specific in vitro or in vivo assays evaluating selected endocrine pathways. For ecotoxicological purposes, there are several different validated in vivo guidelines that utilize mammals, amphibians, or fish. Results indicating activity in initial tests often trigger further in vivo assays that require larger numbers of animals. These assays provide data on adverse effects and endocrine‐relevant endpoints over more extensive parts of the organism's life cycle. Such “higher‐tier” assays are animal‐ and resource‐intensive and technically challenging to conduct. Such studies also need to distinguish endocrine‐specific responses from those that might be a secondary consequence from systemic or general toxicity; often, this is very difficult to elucidate in the absence of whole animal experiments. The need to assess chemicals for endocrine activity coupled with the reliance on in vivo screening and higher‐tier assays is driving the use of large numbers of animals. For example, a recent study (Lagadic et al., 2019) estimated that the plant protection product (PPP) and biocidal product active substances currently registered in the EU would require >190 000 and >1 200 000 fish and amphibians for mechanistic and adversity testing, respectively. This animal use contradicts growing mandates globally, which increasingly demand that safety assessment move to an “animal‐free” paradigm (e.g., Burden et al., 2015; USEPA, 2019). This highlights the need to further consider how the 3Rs—the replacement, refinement, and reduction of the use of animals (Russell & Burch, 1959)—can be applied.

## PURPOSE AND OBJECTIVES

In February 2020, the UK's NC3Rs and HESI (see Supporting Information S1 for more details) collaboratively brought together experts in the field of ED to participate in a workshop centered around the elucidation of estrogenic/androgenic/thyroid/steroidogenesis (EATS) modalities in fish and amphibians. Delegates were selected by the balanced (authorities, academia, industry, HESI, and NC3Rs) organizing committee based on technical expertise, sector, and geographic spread (predominantly Europe and North America). Delegates shared their knowledge and experiences in (a) planning, conducting, and evaluating in vivo studies and (b) developing and applying nonanimal and/or replacement approaches, also known as new approach methodologies (NAMs), for the assessment and identification of environmental ED. The workshop was attended by 51 scientists from Europe and North America, primarily from government and regulatory agencies (33%); the (agro)chemicals, consumer products, biocides, and pharmaceutical industries (25%); contract research organizations (CROs; 16%); and academia (14%). The remainder (12%) were consultants or represented learned societies. Expertise areas covered aquatic ecotoxicology, mammalian toxicology, in silico, in vitro, and modeling sciences. The key objectives were as follows:

▪ Identify the 3Rs challenges and opportunities;
▪ Explore how replacement approaches and/or NAMs (e.g., in vitro, in silico, and early life‐stage embryos) or refined and/or reduction approaches could potentially be used in decision‐making;
▪ Share experiences across sectors and disciplines to identify collaboration opportunities that maximize the impact of 3Rs approaches; and
▪ Identify knowledge gaps that, if addressed, could have a significant impact on the application and acceptance of 3Rs approaches.

Discussions focused on identifying the next steps needed to progress and increase confidence in the use of replacement and refined and/or reduction approaches for EDC identification with the understanding that regulatory agencies may have a need to make decisions based on the current state of the science. The discussion areas covered (1) the current state of the science regarding animal testing, (2) the current state of the science regarding which NAM and/or replacement approaches are available or possible (covering in silico, in vitro, and nonprotected embryo methods (EFSA, 2005; European Commission, 2010), and (3) applying 3Rs approaches in practice. This manuscript, written by the workshop organizing committee in conjunction with a small number of delegates, provides an overview of those discussions, as well as information gathered from delegates prior to the workshop, and the resulting recommendations. This manuscript is not intended as a consensus statement agreed to by all the workshop participants. It represents a summary, prepared primarily by the organizing committee, which we believe to be a balanced reflection of the discussions and a valuable resource to guide progress in the field.

## CURRENT TESTING REQUIREMENTS AND THE NEED FOR PROBLEM FORMULATION

There are variations in approaches to ED identification and assessment dependent on the intended chemical use and where the product is marketed. Currently, the most regulatory advanced strategies exist for PPPs and biocidal product active substances, including the European Chemicals Agency (ECHA)/European Food Safety Authority (EFSA) guidance for the identification of endocrine disruptors and the US Environmental Protection Agency (USEPA) Endocrine Disruptor Screening Program (EDSP; see Table 1) (ECHA/EFSA, 2018; USEPA, 2020). In addition, some non‐pesticidal chemicals that meet potential exposure route requirements are also subject to EDSP testing. This is largely due to potential for environmental exposure and intended biological activity, making potential adverse effects to nontarget organisms a concern. Other substance types, such as human and veterinary pharmaceuticals and industrial chemicals, will have less direct exposure pathways into the environment (e.g., via wastewater). By their nature, pharmaceuticals also have intended biological activity—some specifically with intended endocrine properties in vertebrates (as do some pesticides that target insect endocrine systems). The identification of the potential for endocrine‐mediated effects of pharmaceuticals and industrial chemicals currently tends to rely on (1) the follow‐up of flags identified by their intended mode of action (MoA), (2) findings (e.g., reproductive effects) within standard chronic testing (particularly in mammals), (3) potential issues identified by read‐across and/or identification of structural similarities, or (4) information from open literature studies.

**Table 1:** Overview of major geographical testing requirements using fish or amphibians for ED identification and assessment across industry sectors

Region	Substance type	Legal basis	Requirements for endocrine testing using fish or amphibian models
Europe	Plant protection products	Commission regulation (EU) 2018/605 amending Annex II to Regulation (EC) No. 1107/2009 by setting out scientific criteria for the determination of endocrine‐disrupting properties	OECD CF (OECD, 2018b) toolbox adopted.
			The ED assessment comprises two tiers (ECHA/EFSA, 2018):^a^
			(1) an assessment of the mammalian data set. (2) if it cannot be concluded based on point 1 that the substance has ED properties, then the generation of new information on fish and amphibians shall be considered.
	Biocidal products	Commission Delegated Regulation (EU) 2017/2100 setting out scientific criteria for the determination of endocrine‐disrupting properties pursuant to Regulation (EU) No. 528/2012	Specific studies in fish may include a MEOGRT (OECD TG 240) or a fish life cycle study covering all the “estrogen, androgen, or steroidogenesis‐mediated” parameters foreseen to be measured in OECD TG 240. Specific studies in amphibians may include a LAGDA (OECD TG 241). These studies do not need to be performed if endocrine activity is sufficiently investigated (i.e., a test according to OECD TG 229/230 and OECD TG 231 is available) and there is no indication that the substance has endocrine activity or effects potentially related to endocrine activity.
	Industrial chemicals	Regulation (EC) No. 1907/2006 concerning REACH	EDs identified as SVHC or equivalent level of concern (see Article 57(f)) are included in the candidate list and are subject to authorization and/or restriction processes. During 2021, the CARACAL subgroup will discuss revision of information requirements to specifically address ED.
	Human pharmaceuticals	Regulation (EC) No. 726/2004	Currently, the European Medicines Agency's Committee for Medicinal Products for Human Use (CHMP) guideline (CHMP 2006) specifies that environmental risks of certain compounds, including endocrine active, need to be addressed irrespective of exposure. Some outlined guidance on testing approaches is also available in the accompanying Q&A document (CHMP 2010).
			A draft revision of the guidance by the CHMP released in 2018 (CHMP 2018) retains the requirements for tailored risk assessment for endocrine‐active substances and makes more specific recommendations for the mechanism of action‐specific testing, including the following:
			OECD TG 229 fish short‐term reproduction assay. OECD TG 230 21‐day fish screening assay. OECD TG 234 fish sexual development test. OECD TG 240 medaka extended one‐generation test. OECD TG 241 larval amphibian growth and development assay.
	Veterinary pharmaceuticals	Regulation (EC) No. 2001/82/EC	Required studies (VICH 2000; VICH 2004)^b^ do not currently include studies specifically designed to identify endocrine‐mediated effects. Information from mechanism of action, mammalian toxicology, or open literature studies can inform requests for additional studies.
USA	Pesticides, pesticidal formulation inert chemicals, and environmental contaminants found in drinking water to which a substantial population is exposed	EDSP 1998 *Federal Register Notices*	EDSP uses a two‐tiered approach:^a^
			Tier I
		11 August 1998: Endocrine Disruptor Screening Program 28 December 1998: Endocrine Disruptor Screening Program Statement of Policy	OPPTS 890.1100—Amphibian Metamorphosis (Frog) OPPTS 890.1350—Fish Short‐Term Reproduction Assay
			Tier II
			OCSPP 890.2200—MEOGRT OCSPP 890.2300—LAGDA
			52 pesticides on List 1 have been subjected to Tier I screening and no test orders for Tier II testing have been issued as yet. List 1 includes chemicals that the EPA selected based on exposure potential. The EPA is currently focusing on the pivot strategy of developing and implementing NAMs as alternatives. Over time, the goal is to develop a set of “non‐animal” high‐throughput assays and computational bioactivity models as alternatives for all the assays in the current Tier 1 screening battery.
	Human pharmaceuticals	US: National Environmental Policy Act of 1969 Code of Federal Regulations (CFR) Part 25—ENVIRONMENTAL IMPACT CONSIDERATIONS	The US Federal Drug Administration (FDA) has issued both a general guidance for ERA (FDA 1998) and a Q&A for endocrine active substances (FDA 2016). Neither of these request specific studies, but applicants are encouraged to requested to consult the Agency prior to submission.
Japan	Chemical substances	Japan Chemical Substance Control Law (2011)	The Japanese strategy uses a two‐tiered approach:^a^
		Current strategic program EXTEND 2010	Tier I
			Medaka estrogen receptor *a* reporter gene assay. Medaka androgen receptor *b* reporter gene assay. AMA (OECD TG 231). Fish screen (i.e., OECD TG 229 or 230). JMASA (under development).
			Tier II
			LAGDA (OECD TG 241) MEOGRT (OECD TG 240)

Abbreviations: AMA, Amphibian Metamorphosis Assay; CARACAL, Competent Authorities for REACH and CLP; CF, conceptual framework; ED, endocrine disruption/disruptor; EDSP, Endocrine Disruptor Screening Program; EXTEND, Extended Tasks on Endocrine Disruption; JMASA, Juvenile Medaka Anti‐androgen Screening Assay; LAGDA, Larval Amphibian Growth and Development Assay; MEOGRT, Medaka Extended One‐Generation Reproduction Test; NAM, new approach methodology; OECD, Organization for Economic Cooperation and Development; SVHC, substance of very high concern; TG, test guideline; USEPA, US Environmental Protection Agency.

^a^ Other in vitro and mammalian‐based assays are also required (OECD or USEPA equivalent TGs normally also accepted).

^b^ Also applies to the USA and Japan.

The geographical differences in approach are also marked in some sectors, noting the risk‐based approach adopted in North America and Japan compared with the hazard‐based approach often favored in Europe. The European hazard‐based approach effectively prevents consideration of exposure in the assessment; although there is a legislative allowance for use under “negligible exposure” conditions, however, for the environment, this has not been defined. Under this approach, a substance shown to be an EDC will not be approved, resulting in it being removed from the market or its use severely restricted, regardless of whether safe uses can be identified.

The data needs and testing requirements for EDC identification and assessment are context dependent, and it is critical that this context is identified and considered upfront via a problem formulation process. The problem formulation step of any human health or environmental safety assessment seeks to define the scope of the assessment to best determine the data, tools, and procedures required to complete the evaluation. This ensures that the assessment is “fit for purpose” and meets the overall (risk) management goal (Solomon et al., 2016) so that the data generated add value to the decision‐making process and support the selection of studies, in line with the 3Rs. It should be noted that although there are variations in geographical approach, ultimately, many products are intended for wide geographical or global use. Testing requirements or test packages will therefore usually be designed to ensure that all the likely relevant global requirements are fulfilled.

In 2012, the Organization for Economic Cooperation and Development (OECD) published Guidance Document (GD) 150 on Standardised Test Guidelines for Evaluating Chemicals for Endocrine Disruption; this framework provides a guide to the tests available, rather than setting out a testing strategy (OECD, 2012). GD 150 was updated in 2018 to reflect the newer and revised OECD test guidelines (TGs) and to capture scientific advances in the use of test methods and assessment of the endocrine activity and endocrine‐mediated effects of chemicals (OECD, 2018b). The OECD conceptual framework (CF) (OECD, 2012) describes the available (validated and those anticipated to be validated) assays across five “levels”: (1) existing data and/or nontest information, (2) in vitro assays providing data about selected endocrine mechanism(s) or pathway(s), (3) in vivo assays providing data about selected endocrine mechanism(s) or pathway(s), (4) in vivo assays providing data on adverse effects on endocrine‐relevant endpoints, and (5) in vivo assays providing more comprehensive data on adverse effects on endocrine‐relevant endpoints over more extensive parts of the organism's life cycle. The framework is not a testing strategy, but is a toolbox that can be entered and exited at any level depending on information needs and the regulatory framework in which an evaluation is performed. OECD GD 150 (OECD, 2018b) has largely been used as a basis to inform the development of endocrine‐specific data requirements or GDs (such as those highlighted in Table 1) for the implementation of the scientific criteria. Some of these came into force recently (e.g., for PPPs in the European Union [ECHA/EFSA, 2018]) or are anticipated in the near future (e.g., amendments to the European Union's Registration, Evaluation, Authorisation and Restriction of Chemicals [REACH] regulation annexes; updates to the European Medicines Agency [EMA] guideline on the environmental risk assessment of medicinal products for human use). Not only are there variations in the specific tests required under the different chemical regulations, but there are also differences both in how weight‐of‐evidence (WoE) considerations are applied, where a combination of evidence from several sources is considered, and in how open literature is used (Suter et al., 2020).

**Figure 1:**
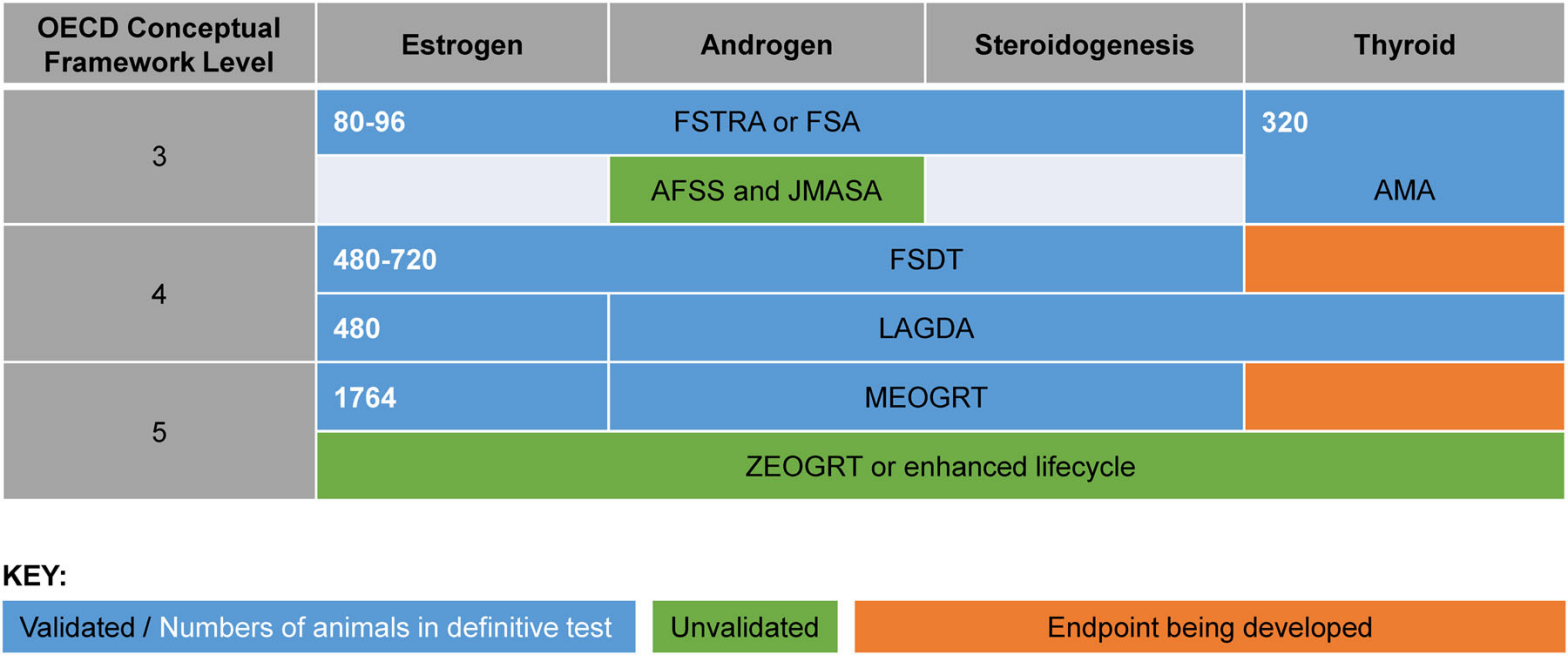
Overview of OECD TGs and standardized test methods available, under development, or proposed that can be used to evaluate chemicals for activity and/or disruption, updated based on the OECD CF for Testing and Assessment of Endocrine Disrupters (OECD, 2012), including an overview of the modalities that each assay is intended to identify (individual regulatory policies may differ on specifics), the TG validation status, and an indication of the number of animals used per test. The CF is intended to provide a guide to the tests available that can provide information for ED assessment, but it is not intended to be a testing strategy. Further information on the use and interpretation of these tests is available in OECD GD 150. Not all the tests are necessarily requested, but OECD CF Level 3 positives may be a trigger for Level 4/5 studies. Note that, under the current EDSP, LAGDA data are considered primarily to identify interactions with only the HPT axis. AFSS, androgenized female stickleback screen (OECD GD 148; variant of OECD TG 230); AMA, Amphibian Metamorphosis Assay (OECD TG 231/OPPTS 890.1100); EDSP, Endocrine Disruptor Screening Program; FSA, Fish Screening Assay (OECD TG 230); FSDT, Fish Sexual Development Test (OECD TG 234); FSTRA, Fish Short‐Term Reproduction Assay (OECD TG 229/OPPTS 890.1350); GD, guidance document; JMASA, Juvenile Medaka Anti‐androgen Screening Assay; LAGDA, Larval Amphibian Growth and Development Assay (OECD TG 241/OCSPP 890.2300); MEOGRT, Medaka Extended One‐Generation Reproduction Test (OECD TG 240/OCSPP 890.2200); OECD, Organization for Economic Cooperation and Development; TG, test guideline;  ZEOGRT, Zebrafish Extended One‐Generation Reproduction Test

The existing and forthcoming requirements across all chemical sectors will have a major impact on the number of animals used in the identification and assessment of environmental endocrine‐mediated effects. This is not only due to the need for additional endocrine‐specific in vivo tests but also because the tests can be complex and highly animal intensive, particularly at the higher CF levels (see Figure 1) (Lagadic et al., 2020).

## POTENTIAL CHALLENGES WITH IDENTIFICATION OF EDCS IN A REGULATORY CONTEXT

Throughout the three workshop discussion sessions, several themes emerged reflecting the current challenges facing EDC identification across all chemical sectors; however, it is acknowledged that not every one of these challenges is applicable to all regulatory EDC identification frameworks. These themes are as follows:

▪ A lack of high‐quality data, or data gaps, for identifying ED, especially where specific tests are not a formal requirement and where there is, by necessity, a stronger reliance on the use of open literature.
▪ The difficulty in establishing the relevance of endocrine effects observed in laboratory studies to the maintenance of wild populations, which is the ultimate protection goal. Currently, there is an assumption that all effects on survival, growth, development, and reproduction will be population‐relevant and the onus is to demonstrate nonrelevance of effects (ECHA/EFSA, 2018). This can lead to some uncertainty as to how assessments might be refined through additional data generation. Endocrine disruption‐relevant adverse outcome pathways (AOPs) are currently limited and are focused more on the first half of the AOP (molecular initiating event [MIE], i.e., endocrine *activity*, and MoA) than the second half (the apical adverse outcome leading to impacts on populations). This is partly related to challenges in linking the MIE with the apical outcome in a scientific, quantitative, and rigorous way (Lagadic et al., 2020).
▪ The complexity of data interpretation, including how to:
  (a) deal with activity and/or effects within the historical control range (e.g., as recommended in the Animal Research: Reporting of In Vivo Experiments [ARRIVE] guidelines) (Kilkenny et al., 2010);
  (b) deal with conflicting data, which can be dependent on the study design and the species used;
  (c) increase confidence in negative results from lower‐tier in vitro studies;
  (d) assign weight to individual results; and
  (e) determine which results are indicative that in vivo testing (for activity and/or adversity) is necessary.

▪ How to distinguish between a primary ED MoA from a secondary MoA when in vivo effects are seen (i.e., when are they endocrine‐mediated vs. caused by systemic toxicity?) (Marty et al., 2018). The main challenge here relates to the setting of the maximum tolerated concentration (MTC) (Hutchinson et al., 2009; Wheeler et al., 2013), which is mainly based on expert judgment. Further, it can be challenging to perform an MoA analysis and establish which modality underlies an impacted parameter, especially within studies in which several pathways are investigated in parallel, an aspect not unique to nonmammalian endocrine studies. This is not an issue under risk‐based programs, where regulation is based on the point of departure established, regardless of whether an ED effect is the primary or secondary toxicity.
▪ The limited understanding of the specific contribution of chemical exposure and the ability to distinguish ED effects from other possible causes (e.g., genetics and environmental factors). This highlights the need for increased relevance of testing strategies.
▪ The gaps within current paradigms to identify effects in nontarget organisms other than fish, amphibians, and wild mammals (e.g., birds and reptiles) or to identify modalities other than EATS.

The following sections discuss the challenges and 3Rs opportunities identified related to the use of (a) in vivo TGs, (b) the application of NAMs, and (c) applying the opportunities in practice.

### Challenges and 3Rs opportunities in in vivo testing

#### In vivo testing challenges

The aim of this discussion session was to deduce where the reduction and refinement opportunities lie, while understanding that, at present, regulatory decisions require data generated from in vivo studies. Assays using nonprotected embryos are considered in the next section on NAMs. Table 1 summarizes the fish and amphibian studies currently required under some regional regulatory programs where in vivo testing is mandated. Figure 1 presents an overview of the modalities that each assay is intended to identify, the TG validation status, and an indication of the number of animals used in each assay. The discussions identified both the advantages and the challenges posed using the currently available in vivo tools; these are summarized in Table 2. Issues related to the use of specific TGs were also raised, although in the interest of brevity, these will be discussed and addressed more thoroughly through future activities.

**Table 2:** Advantages and key challenges identified with generating and using data from currently available in vivo TG assays

**Advantages**
▪ Meets needs to demonstrate adversity at the whole organism level, as determined or required by the current ED definition. ▪ Totality of the whole organism investigated, capturing ADME and complex interactions (feedback and compensatory); sensitive developmental stages may be included. ▪ Increased confidence with increased evidence (i.e., multiple studies in different species, life stages, and concentrations). ▪ For the EATS modalities, relatively broad coverage of TGs, encompassing various known sensitive measures.
**Key challenges**
▪ Complexity: uses large numbers of animals, is expensive, and lengthy timescales are needed to complete and analyze studies. o In addition to the main study, animal use to set up tests can be extensive (e.g., compatibility screening for reproductive groups; FSTRA and MEOGRT F0), and there are issues with test concentration setting finding (see below). ▪ Laboratory capacity and experience currently limited, particularly for OECD CF Level 4/5 studies (cf. MEOGRT and LAGDA) but also OECD CF Level 3 studies (cf. AMA and FSTRA due to high demand in the EU). o There is greater experience in conducting CF Level 3 studies (FSTRA and AMA), although no analysis has been conducted as yet to identify potential for flexibility in current performance criteria, leading to varying regulatory acceptance of different outcomes. Due to limited validation, experience gained with use should inform any potential modifications to increase scientific rigor. Even the OECD TGs indicate that this re‐evaluation should be performed when there is more experience using the test methods (e.g., of MEOGRT and LAGDA). ▪ High variability of key parameters measured (e.g., vitellogenin and reproduction). ▪ Multiple MoAs not differentiated in most studies. o Adverse effects within the same study provide little information on the actual mechanism; if effects are seen in an OECD CF Level 3 study, a higher‐tier study using more animals will usually be required. ▪ Awareness of test limitations needed, since study designs may not be directly relevant to environmental scenarios (i.e., some designed explicitly not to be), particularly since there is divergence in scientific opinion as to how important these issues are for robust regulatory evaluations. o They may not capture effects due to short exposure duration, or sometimes, effects will be on a multigenerational scale, very subtle, or occur at lower exposures than those tested. Additionally, there are questions around the relevance of individual test species as surrogates for all environmental species. ▪ Measured endpoint(s) not necessarily representative exclusively of endocrine‐specific effects (e.g., there is an inability to differentiate between primary and secondary endocrine MoA). o Driven by a need for hazard identification, the highest test concentrations feasible are promoted. This increases the likelihood of observing secondary effects that could be mistaken for a direct interaction with the endocrine system. This, coupled with a very low power to assess clinical signs that might be indicative of systemic challenge (e.g., body weight gain and/or body weight loss‐type assessments, as measured in mammals), means that false conclusions may be drawn. ▪ Lacking interpretative guidance. o Sometimes, changes in single endpoints can be highly diagnostic; in other instances, multiple, related endpoints are needed to reliably assess pathway perturbation (e.g., see Ankley & Jensen, 2014). ▪ Concentration level‐setting issues. o Determining maximum tolerated concentrations is problematic (life‐stage and species dependent); extended range finding is often necessary; and there is inconsistency between how this is recommended within different OECD TGs. ▪ Potential for variation in sensitivity depending on species choice (fathead minnow vs. medaka vs. zebrafish), which can be driven by geographical preferences. ▪ Prescriptive nature of current data requirements (little room for maneuver away from the default).

Abbreviations: ADME, absorption, distribution, metabolism, and excretion; AMA, Amphibian Metamorphosis Assay; EATS, estrogenic/androgenic/thyroid/steroidogenesis; CF, conceptual framework; ED, endocrine disruption/disruptor; FSTRA, Fish Short‐Term Reproduction Assay; LAGDA, Larval Amphibian Growth and Development Assay; MEOGRT, Medaka Extended One‐Generation Reproduction Test; MoA, mode of action; OECD, Organization for Economic Cooperation and Development; TG, test guideline.

#### Reduction and refinement opportunities

Although largely considered to be the “gold standard” and offering distinct advantages, the challenges identified in Table 2 clearly demonstrate that the currently available in vivo methods are not perfect. There are several opportunity areas to build on best practices, which could reduce the number of animals used or increase the utility of information gained by improving existing study design. It is critical to (a) consider upfront whether fish and amphibian data are needed to answer the regulatory question (acknowledging current regulatory requirements); (b) ensure that the utility of the data generated is maximized and adds value to the assessment process; and (c) ensure that any additional measurements incorporated into assays do not impact the welfare of the test animals. The first aspect relates to the need for problem formulation, and there is scope to consider whether data from other study types and species can provide the information on which to base a regulatory decision. Many opportunities could be harnessed through the exploitation of existing flexibility or the introduction of greater flexibility within legal frameworks or TGs (while noting that this could contribute to uncertainty for registrants). This could include more options as to which assays are used to meet specific information requirements depending on the preexisting information. In addition, it is recognized that there is some degree of overlap between certain assays, and this redundancy may be considered desirable. Most opportunity areas identified relate to improving the data generated and its interpretation:

(a) Improvements to concentration‐setting guidance and subsequent data analysis or interpretation, which will require an agreed‐upon definition on the MTC and development of an optimized strategy. This will help to separate out effects not specific to endocrine MoAs (e.g., systemic toxicity) and thus increase confidence in the results and maximize the information generated (fewer confounded treatment levels).
(b) Review and update (if necessary) in vivo TGs to better understand study performance and test validity criteria, in terms of which criteria are fundamental to the performance of the test and for which there are acceptable levels of flexibility, particularly for higher‐level studies in the OECD CF. There is currently no set mechanism (e.g., through the OECD) for review of established TGs and assessment of their utility or relevance, although such a review is recommended in both the OECD TGs for the Medaka Extended One‐Generation Reproduction Test (MEOGRT, OECD TG 240) and the Larval Amphibian Growth and Development Assay (LAGDA, OECD TG 241). High‐quality historical control data can be used to better understand the biological relevance of the tests and facilitate understanding of variability within a method; this will be critical when evaluating new approaches (e.g., NAMs) that may be alternatives to in vivo TGs in the future. Note, variability within the methods has at least two components: (1) intrinsic biological variability and (2) controllable experimental variability (organism source, husbandry, equipment, and personnel) (Brooks et al., 2019). A better understanding of historical controls can also assist with interpretation issues, ensuring the best use of in vivo test data (Wheeler et al., 2019).
(c) Ensure that tests better address population relevance, which may be key for their regulatory use, and that appropriate extrapolations from laboratory to field can be made. There would be utility in identifying appropriate measures that support extrapolation to the population level in a quantitative manner (e.g., through the development and use of quantitative AOPs).
(d) Improving the application of WoE approaches, including better utilization of existing data and greater consideration of other vertebrate data, such as from mammals (McArdle et al., 2020). Decisions to categorize a chemical as an ED should not be made based on data from one in vivo TG alone, and clear WoE guidance and approaches are needed. Under EDSP, the USEPA accepts Other Scientifically Relevant Information (OSRI) submissions in response to test orders; this may include open literature and NAM data (USEPA, 2009). The wider use of minimum reporting requirements, such as criteria for reporting and evaluating ecotoxicity data (CRED) (Moermond et al., 2016) and ARRIVE (Percie du Sert et al., 2019), will aid in transparency and could help facilitate better availability of data and their integration into assessments. Mammalian‐based activity data can also potentially better inform other vertebrate assessments. However, the differences in the toxicokinetics (TK) of different exposure routes (e.g., oral gavage or dietary with first‐pass metabolism in mammals vs. aquatic exposure over the gill in fish) should be addressed as an uncertainty and highlights the need for a better understanding of internal exposure differences across species. The collation of case studies demonstrating real‐life examples of how data from different sources can and have been integrated to inform regulatory decision‐making would be beneficial.
(e) Adding value to existing tests through the addition of endpoints (e.g., thyroid measurements in fish) and links between activity and adversity within the same study could reduce the number of different studies required. However, it should be noted that this could further confuse data interpretation and cause statistical issues if too many endpoints are included in the same test. There are also limitations on availability of plasma and tissue samples for endpoint evaluation (e.g., a fish liver can be used for vitellogenin mRNA or histopathology, but not for both analyses).
(f) More emphasis on developing improved screening tools to decrease the number of chemicals requiring in vivo testing. There is a need to establish confidence in lower‐tier in vitro negatives to avoid triggering unnecessary *higher‐tier* studies, including gaining a better understanding of false positives and false negatives.

### Challenges and 3Rs opportunities in NAMs

#### Challenges in NAMs

The aim of this discussion session was to explore the current status of NAMs for regulatory decision‐making and identify where the opportunities lie for replacing (or reducing) in vivo TG studies. A range of approaches are available, varying in speed of throughput and physiological relevance, from computational and in silico models, in vitro assays using immortalized cells or primary tissues or cells, through to assays using nonprotected embryos. It should be noted that in many regions, embryos, until they reach the free‐feeding life stage, are not protected under animal welfare legislation in the same way as juvenile and adult fish and amphibians (European Commission, 2010). At this life stage, embryos are considered in capable of experiencing pain, distress, suffering, or lasting harm (EFSA, 2005) and are considered a NAM. For a list of NAM examples, see Table 3. For now, NAMs are only used to provide supporting information within data packages. However, NAM data are starting to be considered within a regulatory context as follows:

▪ In vitro consensus approaches have been proposed by the USEPA for estrogen receptor (ER) (USEPA, 2015) and androgen receptor (AR) activity (USEPA, 2017) (so‐called “ER/AR pathway models”).
▪ A proposed integrated approach to testing and assessment (IATA) for the ER has been published (OECD, 2018a; Webster et al., 2019).
▪ Canada's Ecological Risk Classification approach (ERC2) under its Chemicals Management Plan utilizes WoE approaches that incorporate in silico flags.
▪ In November 2020, ECHA/EFSA issued a draft addendum to the GD (ECHA/EFSA, 2018) detailing under which conditions they will accept data from the embryonic *Xenopus* Eleutheroembryo Thyroid Assay (XETA, OECD TG 248) for PPP and biocide registrations.
▪ The output from the ToxCast ER bioactivity model is accepted under the EFSA/ECHA guidance (ECHA/EFSA, 2018) as a replacement for the uterotrophic bioassay in rodents (OECD TG 440, CF level 3).

**Table 3:** Examples of available NAMs for use in EDC identification

Type	NAM	Description
Computational and in silico models	Consensus QSAR models for ER and AR activity	ER: CERAPP project AR: CoMPARA project
	Cross‐species extrapolation models (SeqAPASS) and Automated Approach for Assessing Protein (Molecular Target) Similarity	SeqAPASS (LaLone et al., 2016)
	ToxCast ER and AR pathway models	Integrates the results of multiple in vitro assays providing comprehensive coverage of the pathway (Judson et al., 2015). In the EU, or human health assessment, can be used in place of the uterotrophic assay (ECHA/EFSA, 2018; USEPA, 2015). AR model (Kleinstreuer et al., 2016) as for the ER model, but less developed and not currently considered equivalent to in vivo assays. In the US, considered for determinations of “endocrine disruption screening.” USEPA has proposed the ER pathway model as an alternative to the ER in vitro assays and the uterotrophic assay (USEPA, 2015), and the AR model (Kleinstreuer et al., 2017) has been subjected to extensive peer review.
In vitro assays	ToxCast	ToxCast contains 18 different high‐throughput assay technologies measuring different points along the ER signaling pathway (Judson et al., 2015), plus AR‐related assays (Kleinstreuer et al., 2017), H295R steroidogenesis (Karmaus et al., 2016), and multiple thyroid assays.
Embryo assays	XETA	Published Test Guideline—OECD TG 248
	REACTIV assay	Approved as an OECD project in 2020
	EASZY assay	Draft OECD Test Guideline
	RADAR assay	Draft OECD Test Guideline
Transcriptomic approaches	EcoToxChips	qPCR arrays and data evaluation tool (EcoToxXplorer.ca) for the characterization, prioritization, and management of environmental chemicals and complex mixtures of regulatory concern.
	Transcriptomic dose–response modeling	To establish whether the lowest dose to induce significant transcriptomic changes corresponds to the safe long‐term exposure dose.

Abbreviations: AR, androgen receptor; CERAPP, Collaborative Estrogen Receptor Activity Prediction Project; CoMPARA, Collaborative Modeling Project for Androgen Receptor Activity; EASZY, Endocrine Active Substance, acting through estrogen receptors, using transgenic cyp19a1b‐GFP Zebrafish embrYos; ECHA, European Chemicals Agency; EDC, endocrine‐disrupting chemical; EFSA, European Food Safety Authority; ER, estrogen receptor; NAM, new approach methodology; OECD, Organization for Economic Cooperation and Development; qPCR, quantitative PCR; QSAR, quantitative structure–activity relationship; RADAR, Rapid Androgen Disruption Adverse Outcome Reporter; REACTIV, Rapid Estrogen ACTivity In Vivo; TG, test guideline; XETA, *Xenopus* Eleutheroembryo Thyroid Assay.

Clearly, the tools applied will vary depending on the scientific or regulatory question being posed, in line with the need to consider problem formulation upfront.

The discussions identified both the advantages and the challenges posed using the currently available NAMs for decision‐making: these are summarized specific to assay type in Table 4. Some challenges were identified that are relevant to all NAM types. Participants felt that, currently, NAMs hold most promise for initial chemical screening and prioritization, and there is limited confidence in their use as predictive tools in risk or hazard assessment. This is partly due to a general lack of representation of complex biological processes and systems within the assays (e.g., absorption, distribution, metabolism, and excretion [ADME]). There is a general concern around the risk of false negatives or false positives, and there is a reluctance to make decisions solely based on results from NAMs (although it should be noted that it is also unclear how predictive the in vivo studies are in comparison to the NAMs.) Validation studies are usually conducted using reference compounds, which provide strong negative or positive outcomes. However, most “real‐world chemicals” tested for regulatory purposes will not provide such clear effects, and there is a need to ensure that assays can detect effects across a spectrum of potencies. In general, there are limited NAMs covering key pathways, especially for the thyroid modality, and not all known mechanisms within a pathway are necessarily captured. Further, although it is widely recognized that IATAs will be the way forward for the application of NAMs in practice (through the combination of data from multiple sources), the current OECD process for IATA adoption is rigid, conservative, and lengthy (5–10 years). It typically requires that data are generated using official TG methods, which can take many years to validate, although “performance‐based test guidelines” (PBTGs) are now beginning to be considered; there may be faster ways to enable adoption more broadly within regulatory agencies. Finally, the new approaches are also not necessarily cost‐effective compared to established in vivo approaches, and until there is a stronger regulatory need or mandate for them, they will not be offered as routine services by CROs.

**Table 4:** Advantages and key challenges identified with generating and using data from currently available NAMs

Method	Advantages	Challenges
Computational and in silico models	▪ Rapid and potentially cost‐saving screening methods to prioritize testing of chemicals with the potential to interact with the endocrine system. ▪ Avoids use of highly expensive test materials during early development of new chemicals. ▪ Can be based upon multiple data (in vivo, in vitro, embryonic) on a breadth of to support WoE and reduce reliance on individual assays. ▪ Can be used to prioritize testing (e.g., cross‐species extrapolations can help identify testing needs based on level of conservation of receptors and proteins).	▪ Presently, QSARs can only predict activity and not adverse effects and are not available for more complex exposure and effect situations. Receptor binding models are reliable, but do not indicate the whole organism implications—lack of relevance at the individual and/or population level. ▪ Built based on existing data—the quality of the model is dependent on the quality of the available data. Error from experimental tests is passed along to the model. ▪ Currently parameterized on existing training sets that can be limited in chemical domain breadth compared to other (eco)toxicological endpoints. ▪ Models limited in their design and focus on few key events along the EATS axes. ▪ Currently limited models for thyroid and steroidogenesis modalities. ▪ Use of high‐throughput techniques requires harmonized bioinformatics pipelines and tools.
In vitro and embryo assays	▪ Small in scale, can be used for screening. ▪ Useful starting point if there is a suspected MoA based on analogues particularly for defined pathways with identified MIEs; allows for high‐throughput screening using NAMs focused on particular MIEs rather than investigating every and all MoAs. ▪ Minimizes use of highly expensive test materials during early development of new chemicals. ▪ Useful where in vivo effects have been observed and data on MoA are required to support a WoE. ▪ Quick results, can be cheaper (but not always). ▪ Significant potential to help understand cause and effect at a cellular level and target in vivo testing.	▪ In vitro methods do not generally include metabolism, tissue interactions, or consider the whole organism complexity/life cycle. ▪ IVIVE is needed to understand relevant exposures. ▪ Short‐term studies and exposure duration may not be sufficient to observe endocrine‐mediated effects. ▪ Tests can be expensive, given the resource required to validate results, including analytical support. May need multiple tests to cover one pathway (cf. the ER model [Judson et al., 2015]). Can be difficult to justify the cost, and decisions may be made that money could be better spent on higher‐tier, more definitive studies. ▪ Negative in vitro tests not currently sufficient to demonstrate lack of endocrine activity in vivo in some regions. ▪ Full suite of methods covering all MoAs not yet available. ▪ Assays currently use predominantly mammalian cell lines, which adds to uncertainty regarding relevance to nonmammalian outcomes.
Specific to embryo assays	▪ Captures the complexity of the whole organism, which can include an element of metabolism, and use of a potentially sensitive life stage. Most biological processes are represented at the molecular level (even if physiological activity is not occurring yet). ▪ Can be directly calibrated with apical outcomes from current in vivo TGs and substances can be assessed in the context of relevant endpoints, such as behavior and developmental toxicity, to help elucidate relevance of the endocrine MoA. ▪ Embryonic methods could bridge current in vitro methods and longer duration in vivo methods.	▪ Native genes and/or endpoints available in fish embryos for estrogens and thyroid hormones only. ▪ Androgens accessible via artificial ARE constructs or by cloning the spiggin (stickleback) promotor. ▪ Transgenic models may be too narrow in scope, requiring the use of multiple different models and approaches. As for in vitro assays, can therefore be difficult to justify the cost. ▪ Chorion impermeable to some compounds. ▪ Suite of OECD embryo TGs are expected in the near future (but currently not available). ▪ Questionable whether ADME in embryos is fully relevant to in vivo scenarios.
Transcriptomic approaches	▪ New technologies are enabling rapid testing using multiple endpoint (toxicity pathway) approaches (e.g., gene arrays with 100s to 1000s of targets).	▪ Unclear whether transcriptomic changes can be compensated for and whether they always represent adverse outcomes. Often assessed using tissue from in vivo studies (i.e., animal tests not necessarily avoided).

Abbreviations: ADME, absorption, distribution, metabolism, and excretion; ARE, androgen response element; EATS, estrogenic/androgenic/thyroid/steroidogenesis; ED, endocrine disruption/disruptor; ER, estrogen receptor; IVIVE, in vitro to in vivo extrapolation; MIE, molecular initiating event; MoA, mode of action; NAM, new approach methodology; OECD, Organization for Economic Cooperation and Development; QSAR, quantitative structure–activity relationship; TG, test guideline; WoE, weight of evidence.

#### Maximizing replacement opportunities

The NAM field for ED assessment has come a long way in recent years (Scholz et al., 2013), although there are still fundamental biology questions that remain unanswered and there is a general lack of confidence in the interpretation and use of NAM data on a wider scale. This presents several opportunity areas to either fill knowledge gaps or enhance integration and applicability within current testing paradigms toward truly tiered approaches:

(a) Expanding on the tools available and increasing coverage of biological domains. It would be useful to “map” the tools currently (or soon to be available) in the molecular realm to identify knowledge gaps and inform targeted assay development where needed. Such mapping exercises should lay out the NAMs along endocrine pathways to demonstrate the aspects that they address within an AOP (MIE, key events, etc.) and highlight which aspects are currently lacking. This work is currently underway via multisector teams led by HESI and NC3Rs. The thyroid modality remains an area where, despite the development of numerous NAM assays for MIEs, there are no validated NAM TGs. Transcriptomics data are becoming more available, although better guidance is needed on how to use such data and infer pathways and adverse effects; there is also a need to compare points of departure in traditional tests with transcriptomic points of departure on a wider scale. Greater investment into sequencing and annotating genomes would be beneficial and could support efforts to extrapolate across species and increase understanding of differences in species or taxonomic sensitivity (LaLone et al., 2016), which could then be better captured within assays or in their interpretation. Oviparous embryo models across multiple fish, amphibian, and bird species will be particularly attractive to expand upon, as they are intact in vivo systems that could help to address the limitations of current cell‐ or tissue‐based in vitro tests. As well as covering more species and increasing their use in test batteries, there is a need to expand existing embryo protocols by integrating mechanistic toxicity information (e.g., through gene fingerprinting to inform toxicity pathways or the wider use of transgenic embryo assays, which are easier to design now with technologies such as CRISPR [clustered regularly interspaced short palindromic repeats]).
(b) Increasing confidence in the use of NAMs and data interpretation. It is critical that training and education around NAMs begin at the university level and engage stakeholders across both research and policy making. New tools should be discussed with regulatory agencies at the earliest opportunity to ensure their utility and ultimate acceptance for decision‐making. This includes clear demonstration of “proof of principle” and more standardized ways of interpreting data, including a harmonized WoE framework. There are currently many different documents that cover aspects of WoE in use across different agencies, including OECD GD 150 (OECD, 2018b), EFSA guidance on the use of the WoE approach in scientific assessments (EFSA, 2017), USEPA WoE guidance on interpreting results of the EDSP Tier I battery of studies and identifying candidate chemicals for additional testing with EDSP Tier II studies (USEPA, 2011), and a USEPA publication titled “Weight of Evidence in Ecological Risk Assessment” (USEPA, 2016). These documents support different regulatory contexts, but combine essentially the same information in different ways. Machine learning and artificial intelligence techniques as well as bioinformatics and sophisticated statistical tools may be beneficial to deal with large data sets and could also be useful in guiding method development. A better understanding of nonspecific effects, particularly for the thyroid modality, would be helpful, as would development of a set of reference compounds to benchmark all NAMs (acknowledging this would be difficult to develop). Case studies that demonstrate the value of different study types, including a combination of formally validated and nonvalidated methods, would be useful in the shorter term to build knowledge and advance understanding, without waiting for the outcomes of lengthy formal processes such as TG and IATA development.
(c) Improving the application of systems toxicology approaches. Investigating ED is no different from examining any type of MoA, and this lends itself to the application of pathways‐based approaches, including the AOP framework. The greatest advantage of this would be to ensure that the key events examined by NAMs translate to an endocrine‐relevant adverse outcome—that is, there is sufficient evidence for causal linkages—and ensure that focus is not just on key events that occur early in the AOP that could result from compensatory mechanisms. The relationship between the key event in question and adverse outcome endpoint should be quantitative, highlighting the utility and need for quantitative AOPs (Perkins et al., 2019). The use of bioinformatics, for example, to combine predictions with ADME and conduct in vitro to in vivo extrapolation (IVIVE) will be critical. Regulatory agencies will need to play a part in the bioinformatics development process; these approaches would also have potential to ultimately support a reduction in the need for range‐finding tests for subsequent in vivo studies. The ER pathway model already being accepted by some regulatory agencies (cf. ECHA/EFSA GD) could be used as a conceptual model for other types of receptors. There remains a significant need to expand on work using mechanistic toxicological information such as gene expression signatures (e.g., current efforts to compare ToxCast or “big 'omics” data with apical outcome data or transcriptomic–apical benchmark dose modeling).
(d) Improving exposure considerations. This includes further development of tools that relate applied and intracellular concentrations to whole organism internal exposures (e.g., through IVIVE) and provision of case studies to demonstrate their potential utility and knowledge gaps. This will help to support calibration of in vitro data against in vivo data and improve knowledge of concentrations at molecular target sites. Such data should also include the complexity of both the timing and duration of exposure that can influence the in vivo outcome. Exposure activity ratios to place high‐throughput screening activity in the context of whole organism exposure could be used as a prioritization tool (e.g., see Becker et al., 2015) and allow better interpretation across species.

## HARNESSING THE OPPORTUNITIES AND APPLYING 3RS APPROACHES IN PRACTICE

In an ideal world, to conclude that a substance is an EDC, adverse effects need to be evident with mechanistic data *in the same study* (i.e., a proven causal link), with organism complexity incorporated. We must recognize that not only is this challenging, but there are also many reasons to rethink traditional testing paradigms. Therefore, it is absolutely critical that there is a high level of confidence that the reduction, refinement, or replacement of currently used in vivo tests provides at least as much, if not more, useful information for use in a regulatory framework. It must also be ensured that an increased reliance on NAMs does not decrease our ability to predict adverse outcomes. This will be aided by careful consideration of the regulatory question (or the problem formulation) to determine the following:

▪ Where in the WoE is more information needed?
▪ How can studies be designed to address that question?
▪ Are more data truly necessary?

**Figure 2:**
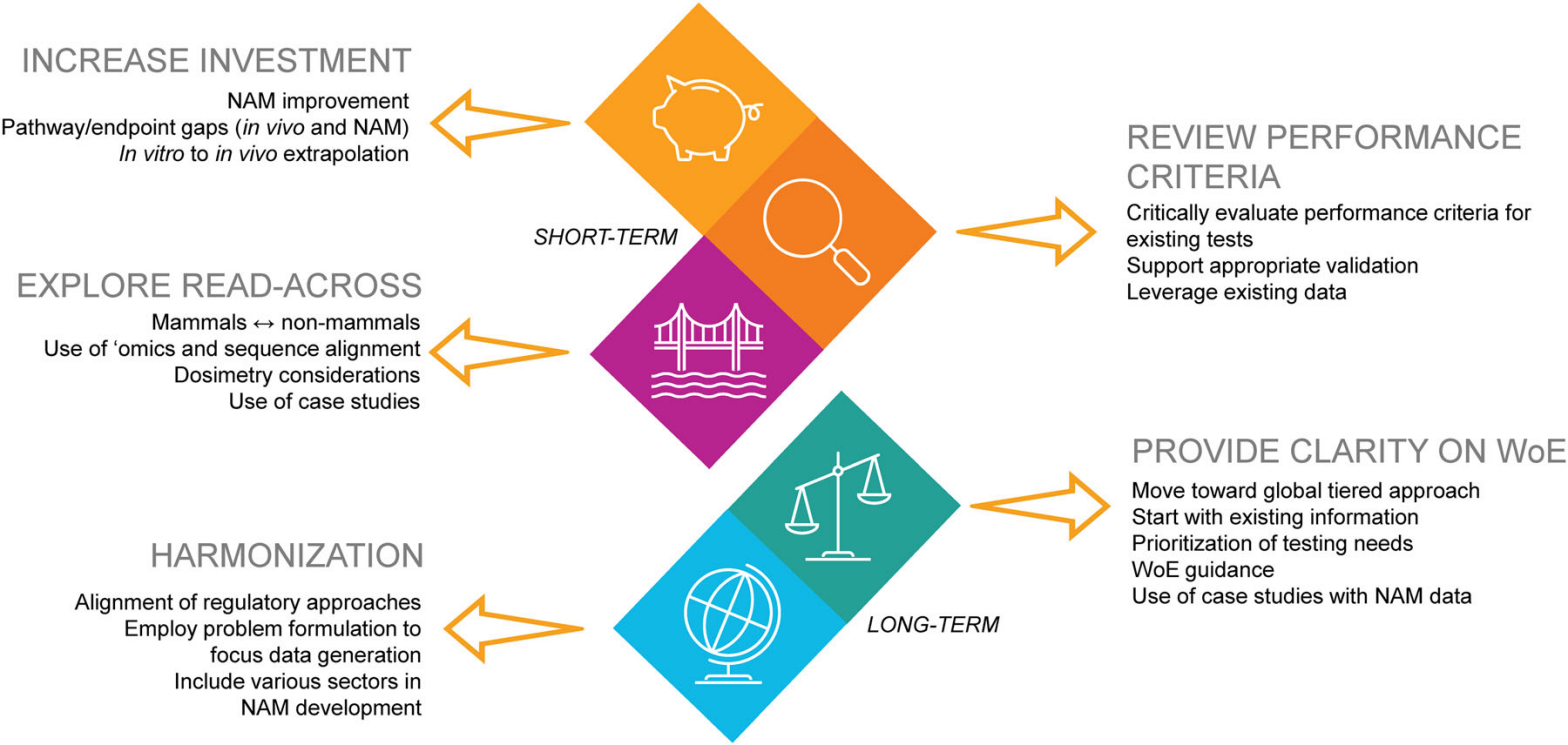
A synthesis of the main workshop outcomes, organized by general topical area as well as projected timeframe

In the longer term, adoption of this way of thinking will support a fundamental paradigm shift that increases the fitness‐for‐purpose of environmental EDC hazard and risk assessment, while harnessing the best and most appropriate science‐driven methodologies. This involves the community acknowledging that regulatory testing cannot capture everything as well as assurance that any testing scheme is environmentally protective. Ideally, there will be a move away from highly prescriptive or broad‐brush approaches toward more flexible ways of operating that identify what is *actually* needed to answer the scientific and regulatory questions. At the same time, the limitations to this shift, including the availability of funding and the capabilities of the available technologies, must continue to be considered. The incentive to reach this goal will be increased if it comes from the top down, as has been the case in other areas of toxicity testing (e.g., activities resulting from the USEPA's announcement to prioritize efforts to reduce animal testing, albeit focused on the use of mammals [USEPA, 2019]). In the meantime, the current workshop discussions have led to the formulation of key recommendations and identification of activities and efforts needed in the short to medium term and the medium to long term to support and inform this paradigm shift, which are outlined below and in Figure 2.

### Short to medium term

*Increase efforts and investment into (a) improving NAMs, (b) addressing pathway and endpoint gaps in both in vivo and NAM approaches, and (c) IVIVE*.

▪ Further develop and standardize in vitro assay batteries that would comprehensively investigate mechanisms within a particular endocrine pathway for activity. Greater incorporation of metabolic competence within in vitro assay test systems.
▪ Conduct a comprehensive review of the current NAM landscape to support identification of research needs. This activity is being initiated by a multistakeholder team led by NC3Rs and HESI.
▪ Provide access to high‐quality in vivo data, including increased understanding of the variability and limitations of animal tests, to both corroborate NAM data and better understand the underlying adverse effects of the MoAs.
▪ Provide adequate funding and allocation of resources to validate and establish which NAMs are best able to reduce or replace and improve in vivo testing. This could include conducting an iterative analysis of the performance of NAMs (making direct comparisons of the conclusions and decisions and hazard or risk assessment outcomes that would be made based on NAM data vs. data from traditional test methods).
▪ Build confidence in the ability of embryo assays to predict adverse effects in laboratory animals by further validating and defining applicability domains using substances that have already been tested in in vivo assays.
▪ Develop better kinetic models to estimate internal exposures and extrapolation from applied doses.
▪ Add more endpoints to existing in vivo tests to maximize their value, provided that this does not cause additional suffering or increase the number of test animals used; examples include ongoing efforts to add thyroid‐relevant endpoints in fish (a focus within the EURION project cluster [Holbech et al., 2020] and OECD Project 2.64: Inclusion of thyroid endpoints in OECD fish TGs).
▪ Build a high‐quality database of historical data of all types, to enable reliable modeling tools to be developed further for the global scientific community.

#### Review performance criteria

▪ Conduct more formal regular retrospective analyses (starting with analysis of historical control data as/when they become available) to evaluate performance criteria and increase awareness of the strengths and limitations of current in vivo methods (e.g., Oris et al., 2012) to (a) increase the value and utility of in vivo test data and (b) to provide a better benchmark for the NAM.
▪ Use retrospective data to better consider validity criteria for in vivo TGs, including which criteria are important and which are less so.
▪ Ensure that there is appropriate ring‐testing during validation phases, as any new test method must be reliably repeatable.
▪ Establish better guidance for concentration setting and interpretation in in vivo tests.

#### Explore the use of read‐across from mammalian data and extrapolation between species

▪ Generation of more information to better enable extrapolation of in vivo information across taxa (for reading across from mammals to nonmammals and vice versa). This includes dosimetry considerations and internal exposure differences, the use of 'omics and/or bioinformatics platforms, and better knowledge of evolutionary genetics and comparative endocrinology.
▪ Greater training and awareness of models such as Sequence Alignment to Predict Across Species Susceptibility (SeqAPASS; www.seqapass.epa.gov/seqapass).
▪ Assimilation of case studies comparing regulatory decisions using mammalian and nonmammalian data. Are both mammalian and fish or amphibian data always needed?

### Medium to long term

#### Provide clarity on giving weight to different approaches and use of existing information

▪ Move toward a tiered approach globally, which starts with existing information and prioritization of testing needs.
▪ Organization of knowledge upfront and better use of the peer‐reviewed and gray literature including incentives to share unpublished data sets (e.g., all data associated with publications [Martin et al., 2019]) and/or establishing a framework to facilitate the sharing of such information.
▪ Development of guidance on assessing overall WoE, including how to weigh different pieces of information: this could be a quantitative weighting matrix to use as a guide. The ERC2 approach developed by Environment and Climate Change Canada is an example of a quantitative WoE framework that could be broadened for use in other jurisdictions and could integrate Bayesian approaches (Bonnell et al., 2018, 2019). This could include development of a “threshold of data” that conclusively enables waiving of higher‐tier in vivo tests and addresses the issue of considering data from multiple NAM sources in place of one in vivo test. It would involve use of in vitro assay batteries to provide a direct indication or link to an adverse outcome at the organism or population level.
▪ Publication of case studies where NAM data have been used for regulatory decision‐making, including how the data were analyzed and synthesized (using a WoE approach if and where appropriate), to demonstrate how each data “type” influences decisions.

#### Improve international harmonization of approaches and their integration

▪ Alignment of regulatory approaches (but not necessarily legislation) based on science both across regions and countries and sector groups, including harmonization of the data required to meet the definition of an EDC, interpretation of whether both activity and adversity data are needed, methods of substance prioritization, and approaches to managing EDCs (risk‐ vs. hazard‐based).
▪ Where different legal frameworks mandate that different data types are generated, use problem formulation to tailor test packages and generate only the necessary and meaningful data (acknowledging that some regional data requirements are more flexible than others). In many cases, companies will be aiming for global marketing and it should be ensured that packages address international needs while reducing duplicative or superfluous testing. Any NAM data generated to meet needs within regional frameworks could be submitted and viewed by all regulatory jurisdictions to help increase confidence in different approaches.
▪ Ensure greater inclusion of different industry sectors and regulatory agencies in NAM development, to support widespread uptake.

## SUMMARY AND CONCLUSIONS

This workshop provided a timely opportunity to bring together experts in the field of nonmammalian vertebrate ED identification and assessment with varying regional perspectives across different regulatory and industry sectors as well as the academic community. The identification and assessment of endocrine properties of chemicals in fish and amphibians under current and forthcoming regional requirements are driving an increase in the number of animals used in regulatory testing, particularly in higher‐tier in vivo studies. It is the ideal time to review the ways in which fish and amphibian in vivo studies are performed and interpreted, to identify where there is scope for improvement, and to assess how NAMs can contribute in this space. The aim is not only to reduce the number of animals used but also to simultaneously improve the science underlying ED assessment.

The workshop discussions highlighted the notable geographical differences in approaches and consequences for substances identified as causing endocrine‐mediated adversity. The needs and requirements for EDC identification and assessment are highly context dependent, and it is critical that this is identified and considered upfront via a problem formulation process, to ensure that only meaningful data are generated and incorporated into decision‐making. Several 3Rs opportunity areas for in vivo testing were highlighted, including addressing the need for improvements to concentration setting and subsequent data analysis and interpretation; reviewing and updating in vivo TGs to better understand study performance and the test validity criteria; ensuring that the tests better address population relevance; improving the application of WoE approaches, including better utilization of existing data and greater consideration of other vertebrate data; adding value to existing tests through the addition of informative endpoints; and placing more emphasis on better screening tools to decrease the number of chemicals needing in vivo testing. Likewise, discussions identified key 3Rs opportunity areas regarding the use of NAMs, including expanding on the tools available and increasing coverage of the biological domains; increasing confidence in their use and data interpretation; improving the application of systems toxicology approaches; and improving exposure considerations. Ultimately, the goal is to stimulate a fundamental paradigm shift that increases the fitness‐for‐purpose of environmental EDC hazard and risk assessment, while harnessing the best and most appropriate science‐driven methodologies. To this end, delegates identified several activities and efforts needed in the short and medium term to support and inform this paradigm change. Two main HESI and NC3Rs multisector, global follow‐up initiatives aimed at addressing the short‐term needs are already underway. The first will review existing in vivo fish and amphibian tests, which are often the “gold standard” tests to which any new alternative methods are compared. This work is focused on analysis of historical control data to evaluate test performance, validity criteria, and strengths and weaknesses. In addition, it will provide an objective but critical discussion of the in vivo tests that will feed into alternative assay development and validation. The second effort is developing a scientific and technical summary of existing or under‐development NAMs that are available to evaluate EATS activity in fish and amphibians. The aim here is to develop a common set of parameters to evaluate NAMs and identify gaps where additional method development is needed. It is acknowledged that some regulatory agencies may have an immediate need to move forward in their decision‐making process, while simultaneously making firm commitments to reduce in vivo testing (e.g., USEPA, 2019). These decisions should be based on the best science available, with re‐evaluations of the state of the science in regular intervals, as necessary. These are first steps, and it is hoped that the wider community engages with the recommendations resulting from the comprehensive workshop discussions and ongoing follow‐up activity and works together to truly benefit the 3Rs in this field, while at the same time increasing the robustness, fitness‐for‐purpose, and relevance of regulatory ED assessments.

## DISCLAIMER

The views and statements expressed in this paper are a reflection of the workshop on “Investigating Endocrine Disrupting Properties in Fish and Amphibians: Opportunities to Apply the 3Rs” (held 25 February 2020 in London, UK) as summarized by the authors. The views or statements expressed in this publication do not necessarily represent the views of the organizations to which the authors are affiliated, and those organizations cannot accept any responsibility for such views or statements.

## Supporting information

This article contains online‐only Supporting Information.

A description of the sponsor organizations and the workshop agenda are included in the Supporting Information.

## Data Availability

All data are presented.
