# Supplementary material for: Investigating endocrine‐disrupting properties of chemicals in fish and amphibians: Opportunities to apply the 3Rs
Source: Integr Environ Assess Manag. 2021 Aug 18;18(2):442–58. doi: 10.1002/ieam.4497 (PMC9292818; doi:10.1002/ieam.4497)
Supplement: Supplementary file 1 — A description of the sponsor organizations and the workshop agenda are included in the Supporting Information. [file IEAM-18-442-s002.pdf]

National Centre for the Replacement Refinement & Reduction of Animals in Research

HESI

NC3Rs/HESI Workshop

Investigating endocrine disrupting properties in fish and amphibians: Opportunities to apply the 3Rs

25 February 2020, central London

9.00 – 9.15	**Registration and refreshments**
9.15 – 9.35	**Introduction, background, and aims of the workshop**<br>*Dr Natalie Burden, NC3Rs and Dr Michelle Embry, HESI*
9.35 – 9.55	**Overview of endocrine disrupter testing, and potential for reducing use of fish and amphibians**<br>*Dr Peter Matthiessen, independent*
9.55 – 10.10	**Pre-workshop regulatory survey results**<br>*Dr Natalie Burden*
10.10 – 10.20	**Introduction to the breakout sessions**<br>*Dr Natalie Burden*
**Breakout session 1:** Current *in vivo* approaches	
10.20 – 10.40	**Overview of current *in vivo* approaches for endocrine disrupting chemical (EDC) identification**<br>*Dr James Wheeler, Shell International B.V.*
10.40 – 11.55	**Breakout discussion** (with refreshments)<br>To cover:<br>▪ How can the current animal tests be optimised to refine and/or reduce animal use?<br>▪ What animal data are needed to address regulatory questions regarding endocrine disruption?<br>▪ What level of confidence is required to avoid higher tier (OECD Level 4/5) animal tests, and how can this be achieved?
11.55 – 12.25	**Feedback from breakout groups**<br>*Rapporteurs*
12.25 – 13.25	**Lunch**

Breakout session 2: Replacement opportunities	
13.25 – 13.45	**Overview of current state of the science – what replacement approaches are available/possible?**<br>*Professor Markus Hecker, University of Saskatchewan*
13.45 – 15.00	**Breakout discussion**<br>To cover:<br>▪ What work is required and/or knowledge gaps filled to enable greater utility and confidence in replacement approaches for scientific and regulatory application?<br>▪ What models/methods are needed to use and interpret these data? e.g., *in vitro* to *in vivo* extrapolation (IVIVE) models, Sequence Alignment to Predict Across Species (SeqAPASS), Quantitative Structure-Activity Relationship models (QSARs)<br>▪ Are there promising approaches under development that should be prioritised for further research?
15.00 – 15.30	**Feedback from breakout groups**<br>*Rapporteurs*
15.30 – 15.50	**Coffee**
**Breakout session 3: Applying 3Rs approaches in practice**	
15.50 – 16.05	**Brief summary of the previous discussions**<br>*Dr Michelle Embry*
16.05 – 17.20	**Breakout discussion**<br>To cover:<br>▪ How could additional replacement approaches be incorporated into weight of evidence arguments/integrated approaches?<br>▪ What are the opportunities for cross-sector harmonisation of requirements?<br>▪ How could mammalian read-across be considered more widely in identifying the need for ecological tests?<br>▪ What are the opportunities to proactively apply 3Rs approaches within new areas of endocrine research and forthcoming regulation?
17.20 – 17.50	**Feedback from breakout groups**<br>*Rapporteurs*
17.50 – 18.00	**Wrap up and meeting close**<br>*Dr Natalie Burden and Dr Michelle Embry*
18.00 – 19.00	**Networking reception including hot buffet**
