# Supplementary material for: Investigating endocrine‐disrupting properties of chemicals in fish and amphibians: Opportunities to apply the 3Rs
Source: Integr Environ Assess Manag. 2021 Aug 18;18(2):442–58. doi: 10.1002/ieam.4497 (PMC9292818; doi:10.1002/ieam.4497)
Supplement: Supplementary file 2 [file IEAM-18-442-s001.docx]

**Supporting information S1:** Background on sponsor organizations

The **NC3Rs** is the UK’s national organisation which leads the discovery and application of new technologies and approaches to replace, reduce and refine the use of animals for scientific purposes and is primarily funded by the British Government. A working group of experts (comprised of regulatory, academic and industry scientists) supports office-based projects that advance the 3Rs in the area of regulatory ecotoxicology. Website: [www.nc3rs.org.uk](http://www.nc3rs.org.uk)

The **Health and Environmental Science Institute (HESI)** brings together scientists from academia, government, industry, and non-governmental organizations (NGOs) from around the world to ensure the health and safety of people and our environment. HESI receives in-kind support for its research programs from participating organizations in the public and private sector and programmatic funding from a combination of government grants and company contributions. As a public charity based in the United States, HESI helps solve the most pressing risk and safety challenges facing humans and the environment today, including ways to lower patient risk, reduce the use of animals in testing, protect the environment, and enhance the safety of products and food we use every day. Website: [www.hesiglobal.org](http://www.hesiglobal.org)
